# Endonuclease G is dispensable for sperm mitochondrial DNA elimination during spermatogenesis in mice

**DOI:** 10.1242/bio.061730

**Published:** 2024-10-07

**Authors:** Xuefeng Xie, Jianshuang Li, Xue Zhang, Shaomei Mo, Ang Li, Tian-Yi Sun, Feng-Yun Xie, Shi-Ming Luo, Guang Wang, Xiang-Hong Ou, Qing-Yuan Sun, Qinghua Zhou

**Affiliations:** ^1^ Guangdong Second Provincial General Hospital, Postdoctoral Research Station of Basic Medicine, School of Medicine, Jinan University, Guangzhou, 510317, Guangdong, China; ^2^ Guangzhou Key Laboratory of Metabolic Diseases and Reproductive Health, Guangdong-Hong Kong Metabolism & Reproduction Joint Laboratory, Reproductive Medicine Center, Guangdong Second Provincial General Hospital, Guangzhou, 510317, Guangdong, China; ^3^ Department of Orthopaedics, Guangzhou Red Cross Hospital, Faculty of Medical Science, Jinan University, Guangzhou, 510220, Guangdong, China; ^4^ The College of Life Science and Technology, Jinan University, Guangzhou, 510632, Guangdong, China; ^5^ International Joint Laboratory for Embryonic Development & Prenatal Medicine, Division of Histology and Embryology, Medical College, Jinan University, Guangzhou, 510632, Guangdong, China

**Keywords:** Maternal inheritance, Sperm, *EndoG*, Mitochondrial DNA, Mitochondrial DNA elimination

## Abstract

Maternal inheritance of mitochondrial DNA (mtDNA) is a widespread phenomenon in eukaryotes. Our earlier research indicated that sperm mtDNA is removed prior to fertilization in mice, and Endonuclease G (ENDOG) orchestrates the degradation of sperm mitochondria in *Caenorhabditis elegans*. However, the mechanisms underlying sperm mtDNA disposal in mammals remain poorly understood. To investigate the potential role of ENDOG in sperm mtDNA elimination, we created *Endog* knockout (*Endog^−/−^*) mice. Our findings revealed that *Endog^−/−^* mice maintained normal spermatogenesis and fertility. Most strikingly, we detected no substantial discrepancy in sperm mtDNA copy number between *Endog^−/−^* and control mice. Furthermore, we noted that sperm mtDNA copy numbers were unchanged in both less motile and motile sperm isolated by Percoll gradient centrifugation from *Endog^−/−^* and control mice. Taken together, our results indicate that ENDOG is not essential for spermatogenesis or the elimination of sperm mtDNA in mice.

## INTRODUCTION

Mitochondria are essential organelles that play critical roles in energy production, metabolism, and signaling in eukaryotic cells. These organelles contain their own genome, known as mitochondrial DNA (mtDNA). However, different from nuclear genomes, mtDNA shows a very dense circular structure and encodes factors critical for oxidative phosphorylation. Mutations in mtDNA have been implicated in various diseases, including mitochondrial disorders, diabetes mellitus, and cancer (Wallace, 1999; Maassen et al., 2004; Ishikawa et al., 2008; Park and Larsson, 2011). In almost all eukaryotes, including humans and mice, mtDNA is exclusively maternally inherited, which is referred to as maternal (uniparental) inheritance (Ankel-Simons and Cummins, 1996; Sato and Sato, 2013). The elimination of sperm mtDNA is recognized as crucial for maternal inheritance across different species (Sasaki and Sato, 2021; Yan et al., 2019), yet the precise molecular mechanisms governing this process in mammals have not been fully elucidated.

Mitochondrial Endonuclease G (ENDOG) is a nuclear-encoded nuclease that is conserved across species (Li et al., 2001). ENDOG was initially considered an apoptotic nuclease that translocates from the mitochondria to the nucleus during apoptosis, mediating chromosomal fragmentation (Parrish et al., 2001, 2003). Additionally, our research and that of others has shown that the ENDOG/CPS-6 (a homologue of the mouse ENDOG protein) is involved in the degradation of sperm mtDNA in invertebrates (Deluca and O'farrell, 2012; Zhou et al., 2016; Chan and Schon, 2012; Baumann, 2016). Moreover, studies in infertile patients have suggested that ENDOG may be a critical factor in the elimination of sperm mitochondria (Eker et al., 2022). However, due to lack of direct evidence, it remains unclear whether the role of ENDOG in sperm mtDNA elimination is conserved in mammals.

Previous research on maternal inheritance has mainly focused on postfertilization mechanisms of elimination of sperm organelles and mtDNA from embryos (Sasaki and Sato, 2021; Song et al., 2016; Rojansky et al., 2016). However, evidence from various species indicates that the pre-fertilization removal of mtDNA from sperm may be a crucial mechanism that contributes to maternal inheritance. In *Drosophila melanogaster*, the elimination of mtDNA before fertilization has been identified as the primary mechanism of maternal inheritance (Deluca and O'farrell, 2012). In the Japanese rice fish (*Oryzias latipes*), sperm mtDNA is progressively reduced prior to fertilization (Nishimura et al., 2006). Our studies, along with others, have demonstrated that the motile sperm already lose their mtDNA before fertilization in mice and human (Luo et al., 2013; Boguenet et al., 2022). Additionally, a recent report in humans has noted that sperm eliminate their mtDNA before fertilization (Lee et al., 2023). Consequently, it is believed that pre-elimination of sperm mtDNA before fertilization serves as a significant mechanism to ensure maternal mitochondrial inheritance in mammals.

In this study, we aimed to determine whether ENDOG is involved in the elimination of sperm mitochondria prior to fertilization. Through the generation and characterization of *Endog* knockout (*Endog^−/−^*) mice, we concluded that ENDOG is not essential for spermatogenesis, male fertility, or sperm mtDNA elimination. There was no significant difference in the average sperm mtDNA copy number between control and *Endog^−/−^* mice, and the motile sperm from *Endog^−/−^* mice showed the same mtDNA copy number as those in the control group. These findings suggest that ENDOG is not required for the elimination of sperm mitochondria.

## RESULTS

### *Endog^−/−^* mice showed normal spermatogenesis and fertility

**Fig. 1.**
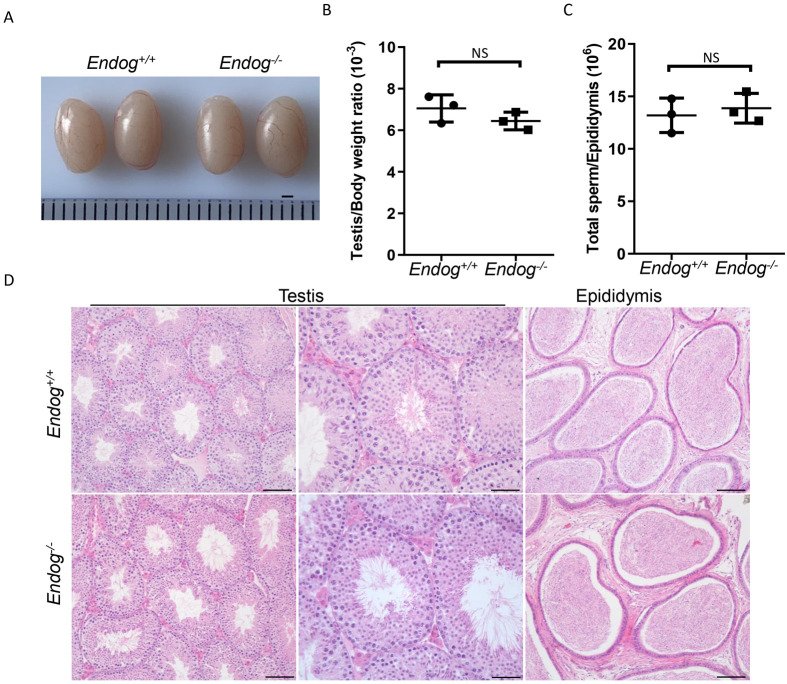
**Spermatogenesis in *Endog^−/−^* mice.** (A) Representative images of testes from *Endog^+/+^* and *Endog^−/−^* mice. Scale bar: 1mm (*n*=3 independent experiments). (B) Testes/body weight ratio of *Endog^+/+^* and *Endog^−/−^* mice (*n*=3 independent experiments). (C) Total sperm number per epididymis from *Endog^+/+^* and *Endog^−/−^* mice (*n*=3 independent experiments). (D) Representative images of testis and epididymis sections of *Endog^+/+^* and *Endog^−/−^* mice after H&E staining (*n*=3 independent experiments). Scale bars: 50 μm. In all of the above experiments, 10-week-old mice were euthanized. The data shown were represented as the mean±s.e.m. Student's *t*-test was performed between *Endog^+/+^* and *Endog^−/−^* mice. NS, no significant difference.

To investigate whether ENDOG is involved in sperm mtDNA elimination in mice, we bred *Endog^−/−^* mice (Fig. S1). The *Endog^−/−^* mice were viable and exhibited no apparent developmental abnormalities. It is known that spermatozoa are produced in seminiferous tubules of testis through spermatogenesis (Griswold, 2016). Given that ENDOG is expressed ubiquitously in all types of germ cells, we assessed the spermatogenesis in the control and *Endog^−/−^* males. The morphology of the testis as well as testis:body weight ratio showed no significant differences between 10-week-old control and *Endog^−/−^* mice (Fig. 1A,B). Similarly, the number of sperm per epididymis in control and *Endog^−/−^* mice was found to be comparable (Fig. 1C). To gain further insight into the spermatogenesis of *Endog^−/−^* mice, we performed Hematoxylin and Eosin (H&E) staining of the testicular sections and epididymides of control and *Endog^−/−^* mice. Both groups exhibited typical seminiferous tubule architecture with the presence of all stages of spermatogenic cells, from spermatogonia to spermatozoa, which is consistent with previous results (David et al., 2006), indicating normal spermatogenesis in *Endog^−/−^* mice (Fig. 1D). Subsequently, we performed fertility test and found that the average number of pups per litter and offspring sex ratios for *Endog^−/−^* male mice was comparable to that of control mice, suggesting that the deficiency of ENDOG did not impact male fertility (Table 1).

**Table 1.**
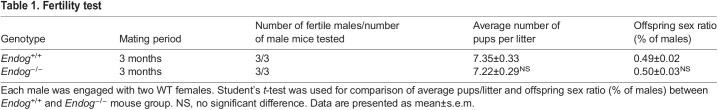
Fertility test

### *Endog^−/−^* mice exhibited normal sperm morphology and motility

**Fig. 2.**
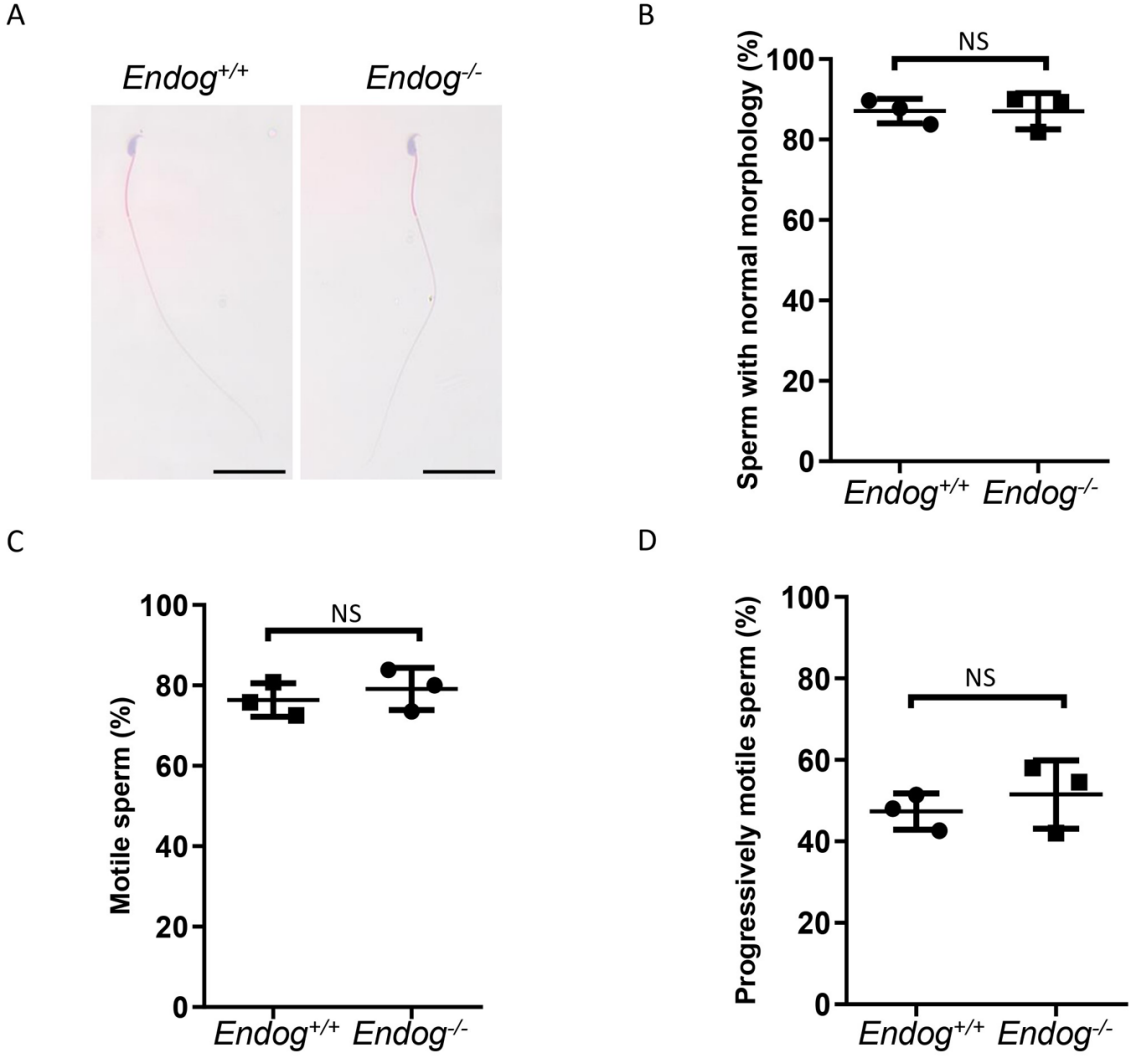
**Sperm morphology and motility in *Endog^−/−^* mice.** (A) Representative images of sperm in cauda epididymides from *Endog^+/+^* and *Endog^−/−^* mice after H&E staining. Scale bars: 50 μm (*n*=3 independent experiments). (B) Percentages of sperm with normal morphology from *Endog^+/+^* and *Endog^−/−^* mice (*n*=3 independent experiments). (C) Percentages of motile sperm from *Endog^+/+^* and *Endog^−/−^* mice (*n*=3 independent experiments). (D) Percentages of progressively motile sperm from *Endog^+/+^* and *Endog^−/−^* mice (*n*=3 independent experiments). In all the above experiments, 10-week-old mice were euthanized. The data shown were represented as the mean±s.e.m. Student's *t*-test was performed between *Endog^+/+^* and *Endog^−/−^* mice. NS, no significant difference.

Considering the association between mtDNA copy number and sperm morphology and motility (Eker et al., 2022), we next aimed to investigate whether the knockout of *Endog* would alter these sperm parameters. We found that epididymal sperm from adult *Endog^−/−^* mice exhibited a normal morphology, which was similar to that of the control group (Fig. 2A,B). Furthermore, we measured the sperm motility, average path velocity (VAP), straight-line velocity (VSL) and curvilinear velocity (VCL) in control and *Endog^−/−^* mice by CASA and observed no significant difference (Fig. 2C,D;  Fig. S2; Movies 1 and 2). These findings suggest that the absence of *Endog* does not affect sperm morphology and motility in mice.

### The mtDNA copy number in the sperm of *Endog^−/−^* mice did not change

**Fig. 3.**
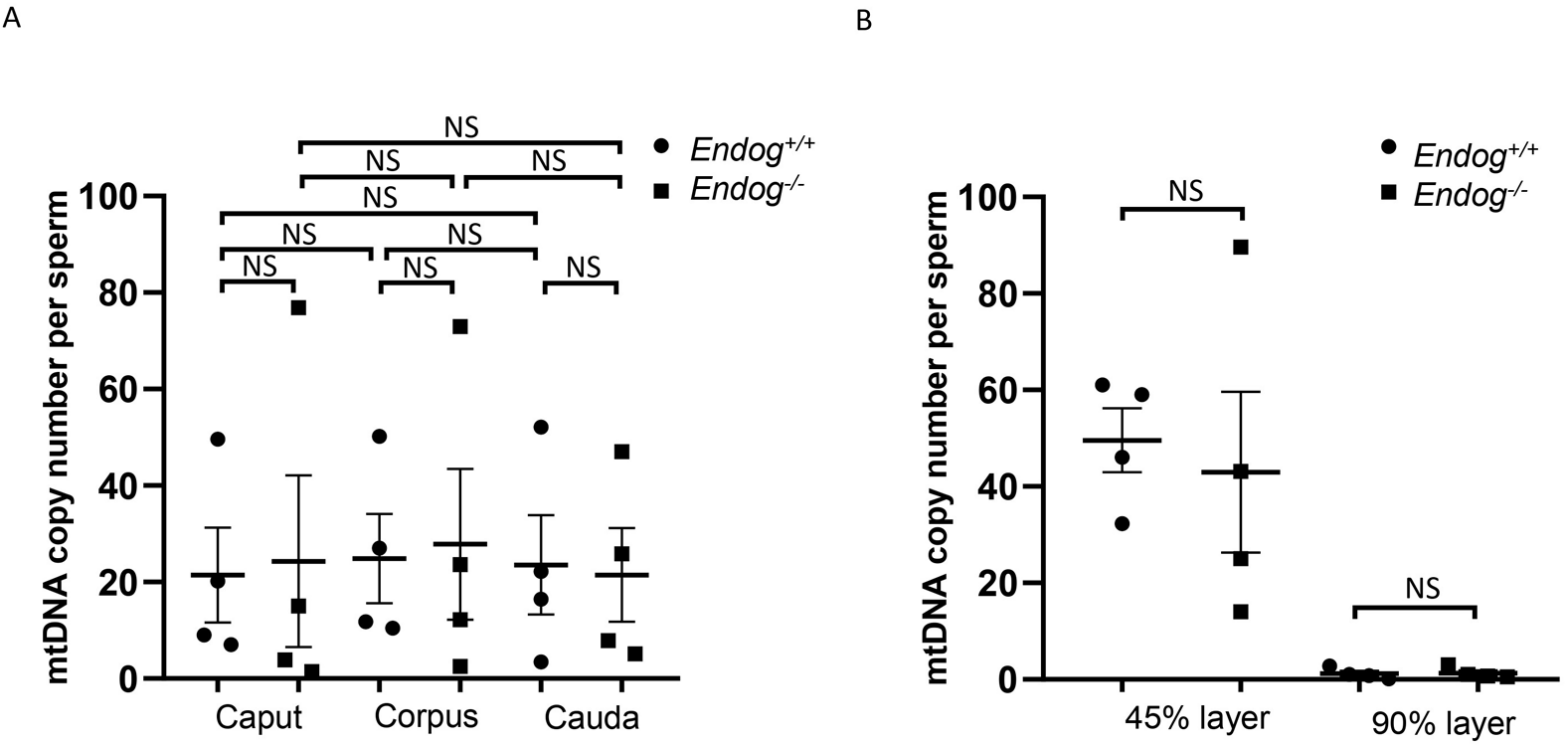
**MtDNA copy number of sperm in *Endog^−/−^* mice.** (A) Quantitative analysis of sperm mtDNA copy number in the caput, corpus and cauda epididymides of *Endog^+/+^* and *Endog^−/−^* mice (*n*=4 independent experiments). (B) Quantitative analysis of sperm mtDNA copy number from 45% and 90% density gradient layers in *Endog^+/+^* and *Endog^−/−^* mice (*n*=4 independent experiments). In all the above experiments, 10-week-old mice were euthanized. The data shown were represented as the mean±s.e.m. Student's *t*-test was performed between *Endog^+/+^* and *Endog^−/−^* mice. NS, no significant difference.

Sperm gain motility as they traverse from the caput (proximal) to the cauda (distal), a process known as epididymal sperm maturation (Nixon et al., 2020). Given the correlation between mtDNA copy number and sperm motility, we speculated that the mtDNA copy number of sperm was altered during epididymal maturation. To investigate this, we used the mtDNA/β-globin ratio to quantify the mtDNA copy number in spermatozoa from the caput, corpus and cauda. We observed the sperm mtDNA copy number from these three regions showed no significant difference (Fig. 3A), suggesting that mtDNA in sperm was not likely to be eliminated during the maturation.

Next, we investigated whether the role of ENDOG in sperm mtDNA elimination is conserved in mice. We compared the mtDNA copy number of sperm collected from various epididymal regions between control and *Endog^−/−^* mice. The sperm mtDNA copy numbers of *Endog^−/−^* mice were comparable to those in the control mice across all regions (Fig. 3A), suggesting that ENDOG is not likely involved in the elimination of sperm mitochondrial DNA in mice.

To further confirm the unnecessity of ENDOG in sperm mtDNA elimination in the motile sperm, we employed Percoll gradient centrifugation to isolate motile and less motile sperm, which typically localize in the 90% and 45% Percoll gradient layers, respectively (Luo et al., 2013). We observed no discernible differences in the mtDNA copy numbers between less motile and motile sperm from both control and *Endog^−/−^* mice (Fig. 3B). Altogether, these data strongly demonstrate that ENDOG is not required for the elimination of sperm mitochondrial DNA in mice.

## DISCUSSION

The present study aimed to investigate the role of the mitochondrial endonuclease ENDOG in the elimination of sperm mtDNA in mice. Knockout of the *Endog* did not affect mouse fertility. The *Endog^−/−^* mice exhibited normal spermatogenesis, and their sperm count, morphology, and motility were remained unaffected. Additionally, the sperm mtDNA copy number from *Endog^−/−^* mice was comparable to that in control mice, regardless of the specific regions of epididymis or subpopulations of sperm motility. Collectively, our data demonstrate that ENDOG is not essential for spermatogenesis nor the elimination of sperm mtDNA in mice.

We observed that mtDNA copy number remained consistent throughout epididymal maturation in both *Endog^−/−^* and control mice, suggesting that sperm mtDNA elimination in mice likely occurs prior to sperm entering the epididymis, possibly during spermatogenesis. This observation is consistent with recent studies in mice and humans that sperm eliminate their mtDNA before fertilization (Eker et al., 2022; Luo et al., 2013). Therefore, it is important to investigate the mtDNA copy number changes during mammalian spermatogenesis to determine the precise timing of mtDNA elimination.

EndoG was initially found to mediate the degradation of sperm mtDNA during spermatogenesis in *Drosophila* (Deluca and O'farrell, 2012; Chan and Schon, 2012). Consistently, we showed that, in *Caenorhabditis elegans*, paternal CPS-6 digests the sperm mtDNA and promotes sperm mitochondrial elimination during fertilization (Zhou et al., 2016). However, our analysis of sperm mtDNA copy number revealed no significant differences between *Endog^−/−^* and control mice, and a similar phenomenon was also observed in other tissues of *Endog^−/−^* mice (Irvine et al., 2005). One possible explanation for this discrepancy is that the function of ENDOG may have diverged significantly during evolution, since ENDOG/CPS-6 is not conserved across all species (Fig.S3). Another explanation could be that other proteins, such as mitochondrial transcription factor A (TFAM), which was recently proven to be involved in sperm mtDNA elimination in humans (Eker et al., 2022), may compensate for the absence of ENDOG. Therefore, it is necessary to identify whether the function of TFAM or other proteins can compensate for the absence of ENDOG in the elimination of sperm mtDNA. Moreover, this discrepancy may also highlight the diversity of mechanisms of mtDNA elimination across species.

Interestingly, the disruption of ENDOG did not affect sperm parameters as we observed no significant differences in sperm number, morphology, or motility between *Endog^−/−^* and control mice. This finding is particularly intriguing given that previous studies in humans have linked *ENDOG* copy number with sperm morphology and motility (Eker et al., 2022). These results suggest that the association between ENDOG and sperm parameters may be more intricate than previously understood, and further investigation across various mammalian species is warranted to elucidate this relationship.

In conclusion, our findings demonstrate that ENDOG is not essential for sperm mtDNA elimination in mice, suggesting that the mechanisms governing maternal mtDNA inheritance may vary between mammals and other organisms. Future research should focus on identifying the pivotal factors involved in sperm mtDNA elimination in mammalian species and exploring potential species-specific variations in this process.

## MATERIALS AND METHODS

### Mice

*Endog^−/−^* mice (C57BL/6) were generated using CRISPR/Cas9 technology as described previously (Wang et al., 2021). Mice were properly nourished on a 12 h light:12 h dark cycle, at a room temperature of 22°C±2°C, humidity of 50%±5%, with free access to food and ddH_2_O. All mouse studies were conducted in accordance with the guidelines approved by the Animal Ethics Committee of Guangdong Second Provincial General Hospital (2024-DW-KZ-060-01). The sequences of primers for mouse genotyping are listed in Table S1.

### Collection of spermatozoa

Briefly, male mice at 10 weeks old were euthanized by cervical dislocation, the epididymides were removed immediately, cleared of any fat and excessive connective tissue, and carefully dissected to isolate the caput (proximal segment), corpus and cauda (distal segment). After multiple incisions were made by curved scissors, every part of epididymides was placed into modified Biggers, Whitten, and Whittingham media (Dun et al., 2012) (91.5 mM NaCl, 4.6 mM KCl, 25 mM NaHCO_3_, 1.7 mM CaCl_2_·2H_2_O, 1.2 mM KH_2_PO4, 1.2 mM MgSO_4_·7H_2_O, 0.27 mM sodium pyruvate, 44 mM sodium lactate, 5.6 mM D-glucose, 5 U/ml penicillin, 5 μg/mL streptomycin, 20 mM HEPES buffer, and 3 mg/mL BSA) (pH 7.4), and the spermatozoa were gently washed into the medium with mild agitation. All sperm preparations were passed through a 40 μm filter, then subjected to 50 mM Tris·HCl buffer (pH 6.8) at 8°C for 20 min before lysis to eliminate contaminated cells. Motile and less-motile spermatozoa were separated by Percoll gradient centrifugation as described previously in details (Luo et al., 2013; Furimsky et al., 2005). Following collection, spermatozoa were lysed in lysis buffer (50 mM Tris-HCl, 1 mM EDTA, 0.5% Tween-20, 100 mg/ml Proteinase K,10 mM DTT, PH 8.5) to release mtDNA.

### Plasmid construction

The vector used as templates for standard amplification curves of mtDNA and *β-globin* were constructed as follows. The fragments of mtDNA and *β-globin* were amplified from C57BL/6j mice with 2× Phanta Flash Master Mix (Vazyme, P520). The backbone sequence of PcDNA3.1 was obtained by restriction enzyme digestion. After the obtained fragments were electrophoresed on a 1.5% agarose gel and gel extraction, the two fragments were mixed and ligated using a ClonExpress II One Step Cloning Kit (Vazyme, C112) according to the manufacturer's protocol separately. All plasmids were validated by Sanger sequencing. The sequences of primers for plasmid construction are listed in Table S1.

### Measurement of the absolute copy number of mtDNA

Quantitative PCR was used to quantify the copy number of mtDNA as described by us previously (Luo et al., 2013). Sequences of primers are listed in Table S1.

### Western blotting

The spermatozoa from epididymis were washed three times with PBS and lysed in 4× Bolt LDS Buffer (Invitrogen, B0007) and 10× blot buffer (Invitrogen, B0009) containing enhanced protease inhibitor (Proteintech, PR20016). Cell lysates were denatured for 10 min and analyzed by western blotting.

The proteins were separated by SurePAGE 10% polyacrylamide gels (GenScript, M00666) and transferred to 0.2 μm pore size polyvinylidene fluoride membranes (Bio Rad, 1620177) using a Bio Rad vertical electrophoresis and blotting apparatus (Bio Rad). Membranes were blocked using 5% (w/v) nonfat milk in TBST buffer (50 mM Tris, pH 7.4, 150 mM NaCl and 0.5% Tween-20) for 1 h and incubated with primary antibodies diluted in TBST buffer containing 5% nonfat milk at 4°C for 12 h. Following incubation with horseradish peroxidase (HRP)-conjugated secondary antibodies for 1 h. Western blots were quantified using a Bio Rad Imaging System. Primary antibodies used in this study were: anti-ENDOG (Cell Signaling Technology, 4969S), GAPDH (Proteintech, 60004-1-lg). The secondary antibody was HRP-conjugated donkey anti-rabbit IgG (Biolegend, 406401) and HRP-conjugated donkey anti-mouse IgG (Biolegend, 405306).

### Histological analysis

The 10-week-old mice were euthanized by cervical disengagement. As described previously (Xie et al., 2022), testes and epididymides were harvested and fixed overnight in Bouin's solution. Tissues were embedded in paraffin after being dehydrated through a graded series of ethanol, and serial sectioning, followed by H&E staining. Images were captured by Digital Leica DFC700T camera installed on a Leica DM2500 microscope.

### Analyses of mouse sperm count, morphology, and motility

10-week-old mice were sacrificed by cervical dislocation. As described previously (Ma et al., 2023), epididymides were dissected and cut into small pieces in Dulbecco's Modified Eagle Medium (DMEM). After release for 30 min at 37°C in humidified incubator. Hemocytometer was used to evaluate the sperm counts. For sperm morphology, the fixed sperm smear slides were stained with H&E (Phygene, PH0516) according to the manufacturer's protocol. At least 200 spermatozoa from each mouse were examined to determine the percentages of morphologically abnormal spermatozoa. For sperm motility, the epididymis with multiple incisions was placed in human tubal fluid containing 10% FBS at 37°C for 5 min, after which the sperm motility was determined by CASA (Computer Assisted Sperm Analysis).

### Fertility test

Three adult males (10 weeks old) were used for fertility tests. Each male mouse was mated with two 8-week-old wild-type female mice for at least 3 months. The numbers of litters and the numbers of pups per litter were recorded.

### Statistical analysis

All statistical analyses were performed using GraphPad Prism software (Graph Pad Software Inc, San Diego, CA, USA). Experiments were performed at least three times independently under similar conditions. All statistical results are presented as the mean±standard error of the mean (s.e.m.) and was calculated using the two-tailed, unpaired Student's *t-*test in GraphPad Prism version 8.0. *P*-value<0.05 was considered statistically significant.

## Supplementary Material

10.1242/biolopen.061730_sup1 Supplementary information
