# Supplementary material for: Endonuclease G is dispensable for sperm mitochondrial DNA elimination during spermatogenesis in mice
Source: Biol Open. 2024 Oct 7;13(10):bio061730. doi: 10.1242/bio.061730 (PMC11554256; doi:10.1242/bio.061730)
Supplement: Supplementary information [file biolopen-13-061730-s1.pdf]

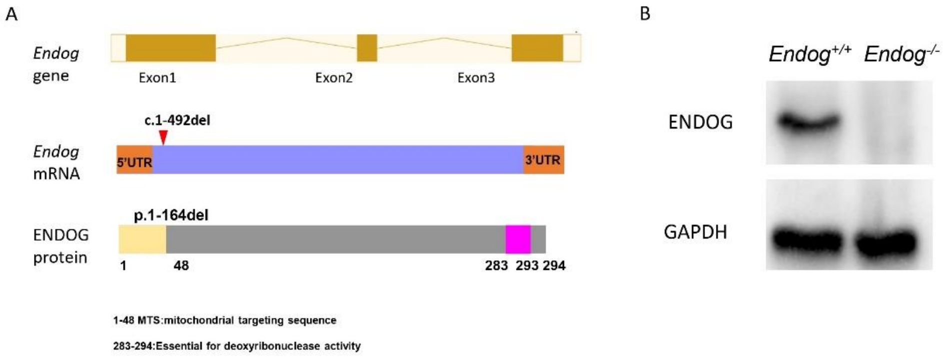

Fig. S1. Generation of *Endog*$^{-/-}$ mouse. (**A**) Descriptive representation of CRISPR/ Cas9 strategy utilized to produce *Endog*$^{-/-}$ mice. (**B**) Western blot of sperm samples confirmed the complete absence of ENDOG in *Endog*$^{-/-}$ mice, while obvious band was seen in control mice. GAPDH was used as a loading control.

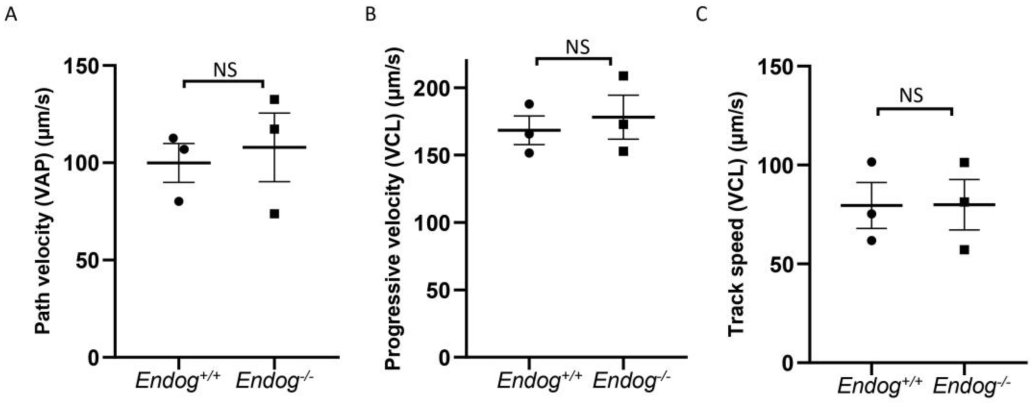

Fig. S2. Velocity parameters of sperm in *Endog*$^{-/-}$ mice. (**A**)The average path velocity (VAP) of the sperm in the *Endog*$^{+/+}$ and *Endog*$^{-/-}$ male mice. (**B**) The average straight-line velocity (VSL) of the sperm in the *Endog*$^{+/+}$ and *Endog*$^{-/-}$ male mice. (**C**) The average curvilinear velocity (VCL) of the spermatozoa sperm in the *Endog*$^{+/+}$ and *Endog*$^{-/-}$ male mice. In all the above experiments, 10-week-old mice were sacrificed. The data shown were represented as the mean ± SEM. Student's t-test was performed between *Endog*$^{+/+}$ and *Endog*$^{-/-}$ mice. NS, no significant difference.

Biology Open • Supplementary information

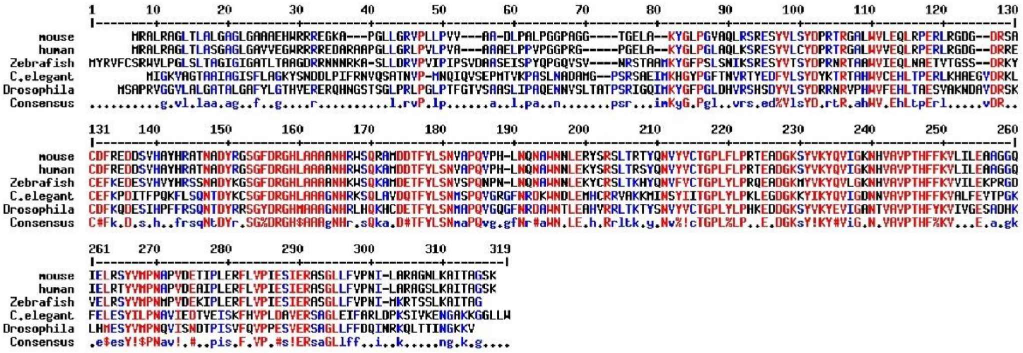

Fig. S3. Alignment of ENDOG from different species. Residues that are identical between different species appear in red and as uppercase letters in the consensus line. Residues highly similar among the above species ENDOG are indicated by red symbols (!, any one of I and V; $, any one of L and M; %, any one of F and Y; #, any one of N, D, Q, E, B, and Z). Unconserved residues are written in blue or as asterisks in the consensus line. The alignment was performed using the online software MultAlin (http://multalin.toulouse.inra.fr/multalin/multalin.html).

Table S1. The primers used in this study

Primer name	Forward Sequence (5'- 3')	Reverse Sequence (5'- 3')
**Primers used for genotyping of the *Endog*$^{-/-}$ mice**		
Endog-F3	TCCTTCACTCTGTGCTAACATC	AACATTTGAAGGACAAGGGCT
Endog-F4	ACTGCAGCGTCTAGATCTTAC	AACATTTGAAGGACAAGGGCT
**Primers used for plasmid construction**		
PcDNA3.1-m-bglobin-F	taatacgactcactatagggagacccaCA AATGTAAGATGGTGCACG	cacactggcggccgttactagtgAA GAACTTGAGGCTTACCCC
PcDNA3.1-m-mtDNA-F	taatacgactcactatagggagacccaAG AGAACTACTAGCCATAGC	cacactggcggccgttactagtgAT CCTCCTTAGTCCTTTAGT
**Primers used for quantification of mtDNA copy number and sperm**		
β-globin Sense Primer	TTCAAGTATACTCACGCCAC	GACATATCTGACATCTCTACTT
Mitochondria Sense Primer	TATCCATCTAGAGGAGCCTG	ATGTAGCCCATTTCTTCCCA

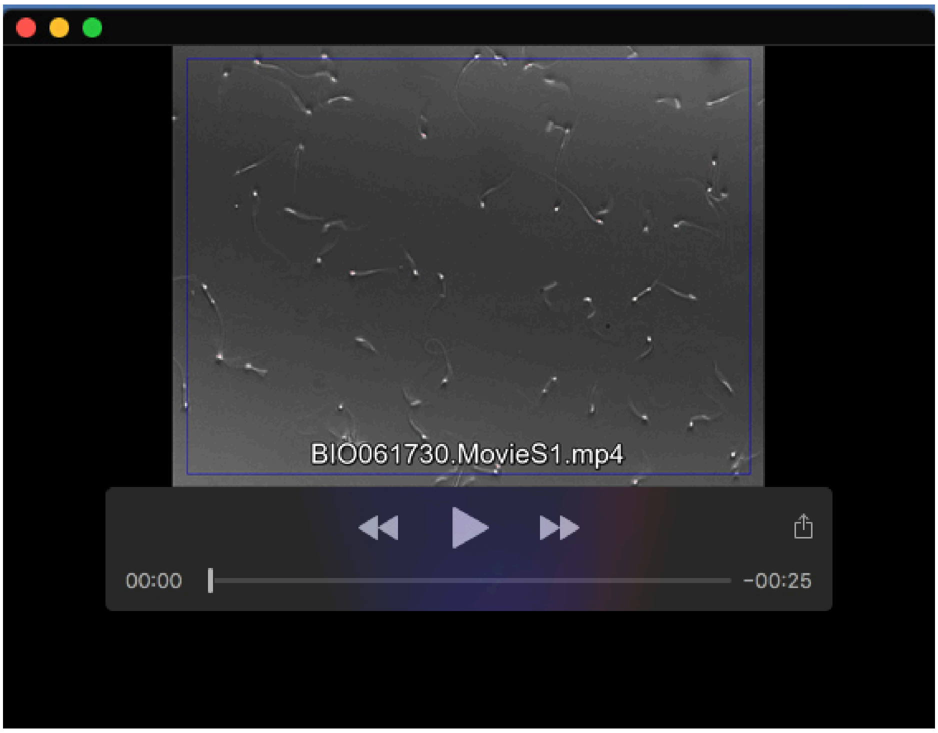

Movie 1. Motility analysis of *Endog*$^{+/+}$mouse sperm.

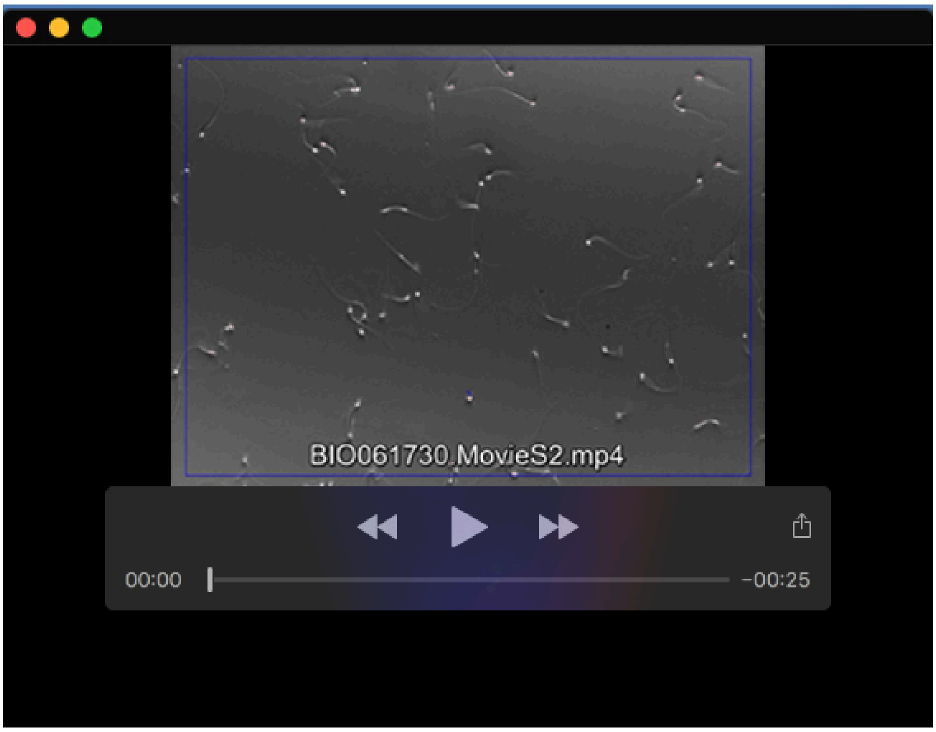

Movie 2. Motility analysis of *Endog*$^{-/-}$ mouse sperm.

Biology Open • Supplementary information
